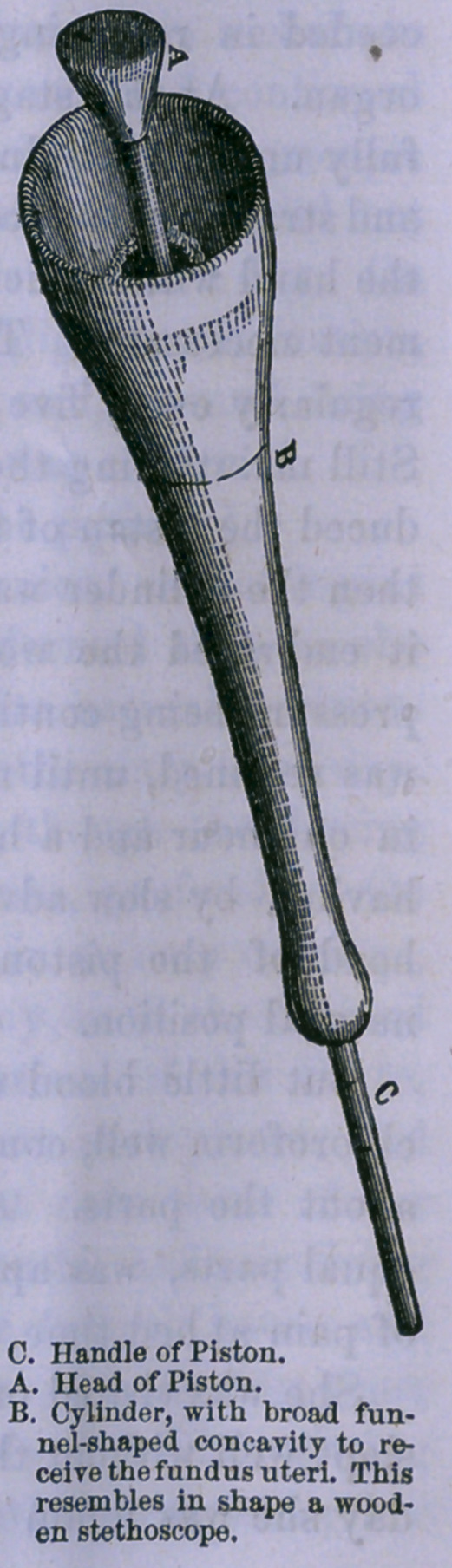# Three Cases of Inversio Uteri Successfully Reduced

**Published:** 1859-06

**Authors:** Hazard A. Potter

**Affiliations:** Geneva, N. Y.


ARTICLE V.

### THREE CASES OF INVERSIO UTERI SUCCESSFULLY REDUCED.

BY HAZARD A. POTTER, M. D., OF GENEVA, N. Y.

REPORTED BY CHARLES N. HEWITT, M.D.

As the treatment of inversio uteri is now attracting the renewed attention of the medical profession, I am induced to report three cases, which have occurred in the practice of my partner, Dr. H. A. Potter. Two of them were cases of complete inversion, and one a case of partial inversion; all of some months' standing.

The first which I shall describe, occurred in the person of Mrs. S ——, of Chicago, Illinois, after the birth of her first child. The patient, aged 21 years, of sanguine temperament, and good physical development, was confined with her first child on the 9th of last April. The labor was not more severe than first labors usually are, and presented no unnatural features, except the occurrence of a severe pain under the right breast, which continued during the three last hours of labor. The child was born at 7 o'clock P. M., and, five minutes after, her physician removed the placenta; and she did not feel any after-pains.

Upon being made aware that the placenta was removed, the patient asked if all was right, and was informed that she must lie still, and let the doctor put the womb back. An attempt to do so caused excruciating pain; and, after some time, all was declared right, and manipulations discontinued.

She passed a sleepless night, and flowed so much that her bed and clothes were saturated. The flowing continued profusely until the fourth day, when she had a severe sinking spell, which lasted two hours and a half, during which time the flowing nearly ceased. She was confined to the horizontal position till the sixteenth day, when, on an attempt to sit up, the hemorrhage again began, and never entirely ceased until the inversion was reduced. She resumed the horizontal position again, and kept it until the twenty-first day, when she was moved, in a chair, to the piano. While there, the flowing returned, and continued profusely until the twenty-fourth day, when it ended in syncope. She now kept her bed until the forty-seventh day, and did not recover sufficient strength to walk until the sixty-third day. On the thirty-ninth day, her physician made a digital examination, and found the womb completely inverted; and, counsel being called on the following day, his conclusion was confirmed.

[Such is the account we received from the patient.]

The patient was then advised to take tonics, use astringent injections, eat nourishing food, and was informed that, as she gained strength, her womb would assume its natural position. This advice was strictly followed, and, by the sixty-third day, she had gained sufficient strength to sit up, though the flowing continued slightly all the while, and, at times, profusely. On the sixty-fourth day, she took a short ride in an easy carriage, and continued to do so on every pleasant day, until she decided to come to one of the hydropathic institutions of this State, where she underwent the treatment usual at such places, and several ineffectual attempts were made at reduction. After remaining at that institution about three months, and perceiving no beneficial result, she went thence to her father's house and sent for Dr. Potter. Dr. Potter visited her, for the first time, on Thursday, the 14th of last October. He found her sitting up; her face and extremities pale and slightly œdematous; her pulse quick and feeble. He made a digital examination. She was flowing slightly at the time. The uterus was completely inverted, and atrophied, being a little smaller than the virgin womb. Its form was nearly cylindrical; its tissues firm and resisting. There was considerable œdema of the lining membrane.

He advised an attempt at reduction, while the patient was under the influence of chloroform. To this she consented. Dr. Potter returned home, had an instrument made, and on Saturday following, performed the operation. I gave the patient chloroform, and, when she was fully under its influence, and in a horizontal position, her hips a little elevated, Dr. Potter began the reduction with the hand. Grasping the uterus firmly, he maintained a steady pressure for twenty minutes, and succeeded in returning the neck, and a part of the body, of the organ. At this stage of the operation, although the patient was fully under the influence of chloroform, she evinced the motion and straining that accompany labor pains, and the vagina grasped the hand with sufficient force to render a resort to the instrument necessary. These contractions of the vagina recurred regularly every five minutes, until the operation was complete. Still maintaining the pressure with the hand, Dr. Potter introduced the piston of the instrument, and applied it to the fundus; then the cylinder was moved upon the stem of the piston, until it embraced the womb; and the hand was withdrawn. The pressure being continued with the piston, every advance gained was retained, until reduction was complete, which was effected in one hour and a half after the operation began. The fundus having, by slow advances, returned within the os, it left the head of the piston with a sensible spring, and resumed its natural position.

But little blood was lost, and the patient rallied from the chloroform well, complaining of nothing but a feeling of soreness about the parts. A folded napkin, wet in alcohol and water, equal parts, was applied to the vulva; and, if she complained of pain at bed time, she was ordered tinct. opii. gtts. xxx.

She was visited on the morning of the second day. She had slept well without the anodyne. On the morning of the third day she was again visited—sat up in an easy chair yesterday afternoon; has walked with ease about the room to-day. There is no pain, and but little tenderness of the parts, and no hemorrhage. She was discharged, and, on the twelfth day after the operation, she took the cars for Chicago.

In calling the attention of the profession to the instrument used in this case, Dr. Potter would not wish to be understood as advising its use in all cases, and under all circumstances in this disease. He believes, as do all who have had experience in the treatment of inversio uteri by reduction, that the hand is the best and safest instrument, when it can be used. This instrument was devised to take its place and perform its duty, and only when the hand could not be employed.

There are cases where the piston alone can be used with great advantage, the hand embracing the womb and directing its movement; and there are other cases where the cylinder will be of great use in place of the hand, as a guide and support to the piston. The entire instrument is made of Turkish boxwood, with the exception of the staff of the piston, which is of hickory.

The head of the piston is of boxwood, and slightly cup-shaped, as far better, for various reasons, than if its upper surface were convex. It is of wood, because, from its slight, adhesive qualities, it is much more readily retained in contact with the fundus of the uterus than either ivory or metal. The cylinder is of wood, because it is lighter, and more readily and cheaply procured than any other substance. The instrument should be boiled in oil before using, to prevent the absorption of blood, or the secretions of the parts to which it is applied. This being an instrument that can be made by any mechanic of ordinary ingenuity, it may be constructed of various sizes to suit individual cases.

C. Handle of Piston.
A. Head of Piston.
B. Cylinder, with broad funnel-shaped concavity to receive the fundus uteri. This resembles in shape a wooden stethoscope.

The other case of complete inversion occurred in 1840. The patient, aged 30 years, was seen by Dr. Potter fifteen months after the inversion, which took place immediately after delivery. For two months, the flowing was profuse, but diminished gradually up to the time of the reduction, when it was scarcely noticeable, except at the menstrual periods. She had leucorrhœa. The uterus was a half-size larger than the healthy unimpregnated womb; sloughy, and yielding to the touch. It was reduced in three quarters of an hour, with the hand alone. The patient suffered greatly during the operation, but made a rapid and complete recovery.

The case of partial inversion occurred in 1836. The patient, aged 35 years, had borne a number of children, and the inversion, when Dr. P. saw her, had existed six months, and she had been treated for polypus uteri. The hemorrhage had not been profuse, but almost constant, and, at the time of the operation, the patient was confined to her bed. Though in the horizontal position, she nearly fainted during the reduction, which was accomplished by the hand in about one half hour. She has borne several children since. The method of practice which insured success in these cases, was, constant, steady, and gradual force applied. The reduction in them all began in the neck, and the fundus was the last portion to pass the os, and passed with the most difficulty.

And we would here inquire, are there any cases of this disease, of however long standing, which may not be reduced by long and continued pressure, excepting only those which are hypertrophied, or in which the peritoneal surfaces are adhered? And, are even these without hope?

## Figures and Tables

**Figure f1:**